# Accuracy of Patient Specific Cutting Blocks in Total Knee Arthroplasty

**DOI:** 10.1155/2014/562919

**Published:** 2014-08-31

**Authors:** Naeder Helmy, Mai Lan Dao Trong, Stefanie P. Kühnel

**Affiliations:** Abteilung für Orthopädie und Traumatologie des Bewegungsapparates, Bürgerspital Solothurn, Schöngrünstrasse 42, 4500 Solothurn, Switzerland

## Abstract

*Background.* Long-term survival of total knee arthroplasty (TKA) is mainly determined by optimal positioning of the components and prosthesis alignment. Implant positioning can be optimized by computer assisted surgery (CAS). Patient specific cutting blocks (PSCB) seem to have the potential to improve component alignment compared to the conventional technique and to be comparable to CAS. *Methods.* 113 knees were selected for PSI and included in this study. Pre- and postoperative mechanical axis, represented by the hip-knee-angle (HKA), the proximal tibial angle (PTA), the distal femoral angle (DFA), and the tibial slope (TS) were measured and the deviation from expected ideal values was calculated. *Results.* With a margin of error of ±3°, success rates were 81.4% for HKA, 92.0% for TPA, and 94.7% for DFA. With the margin of error for alignments extended to ±4°, we obtained a success rate of 92.9% for the HKA, 98.2% for the PTA, and 99.1% for the DFA. The TS showed postoperative results of 2.86 ± 2.02° (mean change 1.76 ± 2.85°). *Conclusion.* PSCBs for TKA seem to restore the overall leg alignment. Our data suggest that each individual component can be implanted accurately and the results are comparable to the ones in CAS.

## 1. Introduction

Since the introduction of knee replacement by Gluck in the 19th century using ivory implants [1], the total knee arthroplasty (TKA) became a reliable treatment for osteoarthritis of the knee. The goal of TKA is to reduce pain and to restore normal function and alignment. Patient satisfaction, complications, implant failure rates, and overall survival rates of primary TKA depend on different factors like medical condition of the patient, choice of implant, and surgical technique. Overall survival rates range from 75 to 90% after 15 years [2]; a satisfactory outcome was shown in 82% with an overall revision rate of 15% after a 10-year follow-up; a satisfactory outcome was shown in 82% with an overall revision rate of 15% after a 10-year follow-up [3, 4].

Multiple studies showed the mechanical alignment as one of the most important factors determining the long-term survival of the prosthesis [5–8]. Recent studies suggest that attaining neutrality in all three coronal alignment parameters is one of the most determining factors concerning patient satisfaction and implant survival. Attaining a mechanical axis and component alignment within a range of ±3° is thought to be associated with a better outcome [9]. Surgical technique and implants developed, different minimally invasive approaches were reported, and computer assisted surgery (CAS) was introduced and has shown to improve the alignment of the prosthesis significantly [10–12]. Potential downsides of CAS are an increased OR time, higher cost, and more surgical steps.

The aim of this study was to determine the accuracy of patient specific cutting blocks (PSCBs) for total knee arthroplasty, designed from a 3D data set. The acquired data is based on CT images of the hip, knee, and ankle and subsequently specific cutting guides are produced by rapid prototype technique for optimal component alignment. We hypothesize that the accuracy of patient specific cutting blocks (PSCBs) is comparable to the one achieved by computer assisted surgery.

## 2. Patients and Methods

We started implanting knee prosthesis with the PSCB technique at our institution in October 2009. Between October 2009 and November 2011, the first 113 patients underwent a primary TKA, using patient specific cutting blocks based on CT data.

Indications for surgery were advanced osteoarthritis, severe pain, and limited function/walking ability.

Excluded from the study were patients without proper preoperative radiographic documentation (*n* = 5) or with previous high tibial osteotomy (*n* = 1) or intraoperative complications not related to the surgical method. One patient died due to a cerebral bleeding after a fall, not related to the surgery, and was lost to follow-up. In seven patients, bilateral surgery was performed at different time points.

Hence, the surgical and radiographic data of 106 patients and 113 knees were collected at a follow-up of three months.

Patients mean age was 69,8 years (range 49–86 years); 66% of the patients were female. We operated on 52 (46%) left knees and 61 (54%) right knees. In one knee, a lateral approach was used due to a severe valgus deformity [13] as it has been shown in literature that, in severe valgus deformities, alignment results are inferior if a standard medial approach is used [14]. In mixed groups (varus and valgus knees), no significant difference in postoperative alignment has been found between medial and lateral approaches [15]. In two cases, an osteotomy of the tibial tuberosity was necessary to expose the joint sufficiently.

In 73 patients surgery was performed by the first author (N. Helmy), a very experienced knee surgeon, in 22 cases by a middle experienced surgeon, and in 18 cases by a resident under supervision of the first author as a teaching operation.

For the measurement of operating time, two patients were excluded due to the necessity of an osteotomy of the tibial tuberosity because this causes a prolonged operating time that does not rely on the PSCB technique. Thus, operating time (time between skin cut and skin closure) was recorded in 111 operations.

From all patients, standardized long-leg standing radiographs and standard knee radiographs (anterior-posterior and lateral views) were obtained pre- and postoperatively.

**Figure 1:**
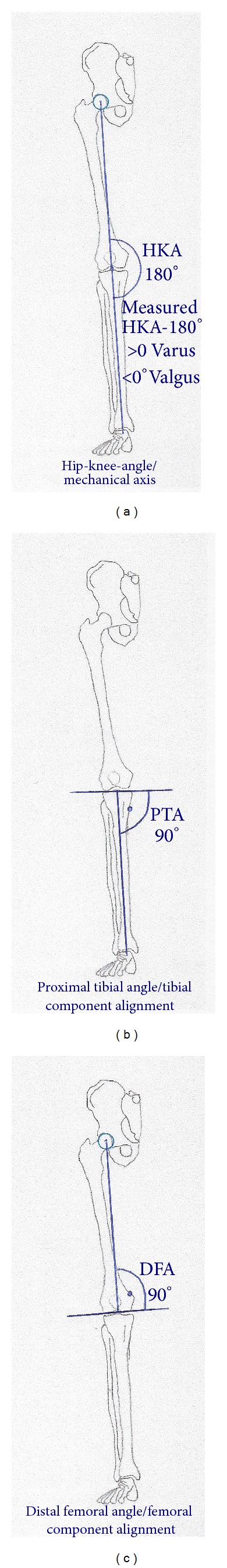
Schematic drawing of the alignment parameters. HKA was defined by the angle between the femoral head centre, the middle of the knee joint, and the middle of the ankle joint (a). DFA was defined by the angle between the femoral head centre, the middle of the knee joint, and a tangential line at the femoral condyles/femoral component in the coronal plane (b). PTA was defined as the angle between the middle of the ankle joint line and a tangential line at the tibial plateau/tibial component in the coronal plane (c).

**Figure 2:**
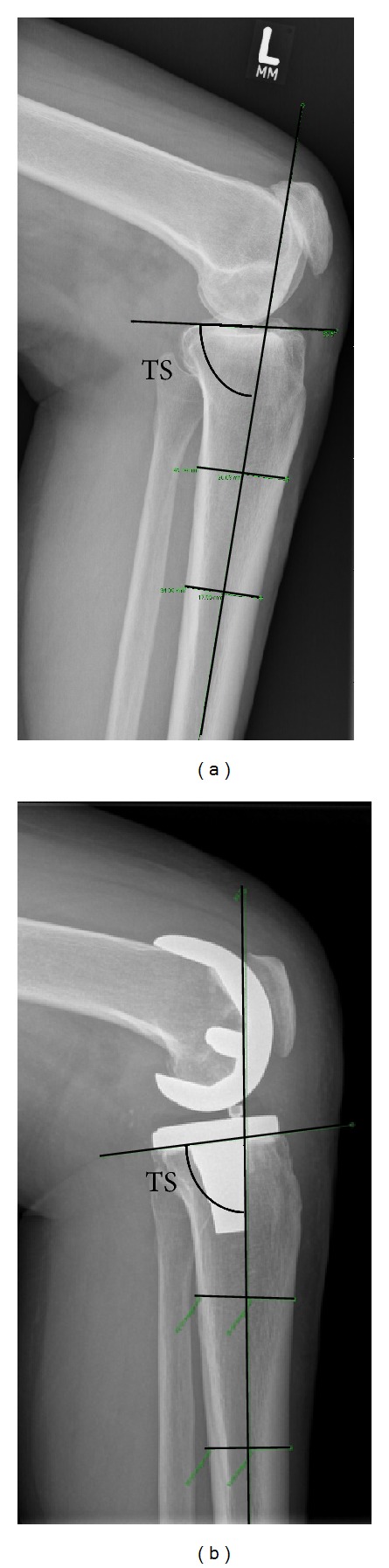
Tibial slope was measured in lateral radiographs of the knee joint preoperatively (a) and postoperatively (b) and was defined as the angle between the proximal tibial anatomical axis and the tibial plateau in the sagittal plane as proposed by Dejour and Brazier [16, 17].

Mechanical leg axis defined by the hip-knee-angle (HKA) and component alignment defined by the distal femoral angle (DFA) and the proximal tibial angle (PTA) were assessed in the coronal plane (Figure 1). Tibial slope (TS) was measured in the sagittal plane [16, 17] (Figure 2). All alignment parameters were recorded preoperatively and at the standard three-month follow-up.

Measurements were taken by an independent surgeon. Each measurement was done three times at different time points and blinded to the name of the patient and the responsible surgeon. Mean values of the three measurements were calculated.

**Figure 3:**
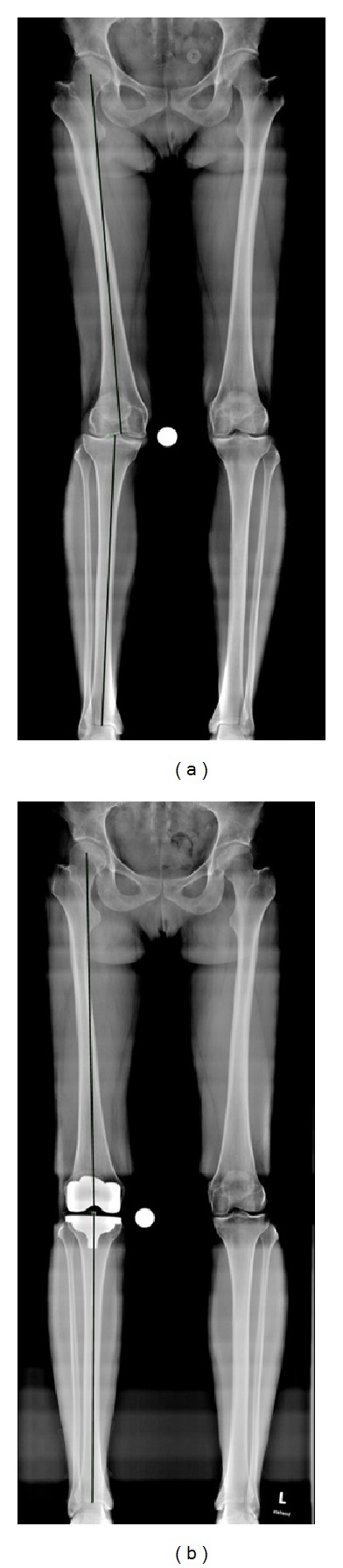
Mechanical leg axis before (a) and after (b) surgery on long-leg standing radiographs.

Calculations and measurements were performed on digitized radiographs using computer software (Centricity^©^) (Figure 3).

### 2.1. Statistical Analysis

Expected ideal alignment for the mechanical axis was 0°. It was defined by the difference between HKA and 180°. Ideal alignment for the femoral and tibial components, DFA and PTA, was 90°. According to current literature, an alignment within 3° of the ideal values (0° for HKA and 90° for DFA and PTA) should be the goal of surgery.

For all patients, the deviation (continuous variables) from the expected ideal alignment and the proportion of patients with deviations ≤1, 2, 3, and 4° from the expected ideal value was calculated.

Concerning the sagittal alignment, maintaining the preoperative posterior tibial slope whenever possible was the goal of surgery. Thus, the difference between the pre- and the postoperative tibial slope was calculated. Sagittal alignment was considered satisfactory if the difference did not exceed 3°.

To describe and quantify the change of the continuous variables between before and after surgery, the difference was calculated and, via inverting a Wilcoxon signed rank test, the corresponding confidence intervals (95% level) were computed [18].

Additionally, we examined whether there is any learning curve concerning accuracy with this technique over the time. To assess this “learning curve,” we provided a plot of the proportion of patients within the 3° tolerance postoperatively, split by each four months. For each quarter, a Wilcoxon confidence interval was added.

### 2.2. Surgical Technique

Implants used were GMK TKA (Global Medacta Knee, Medacta, Switzerland) and posterior stabilized implants were used in all cases.

The medial parapatellar approach and the subvastus approach (Figure 5(a)) were utilized for neutral and varus knees and the lateral parapatellar approach for severe valgus knees (*n* = 1). As the PSCBs are manufactured depending on the approach, the surgeon must decide preoperatively whether he performs a medial or a lateral approach.

After performing the arthrotomy, Hoffa's fat pad is resected partially, ligament release is done, and redundant soft tissue is removed.

**Figure 4:**
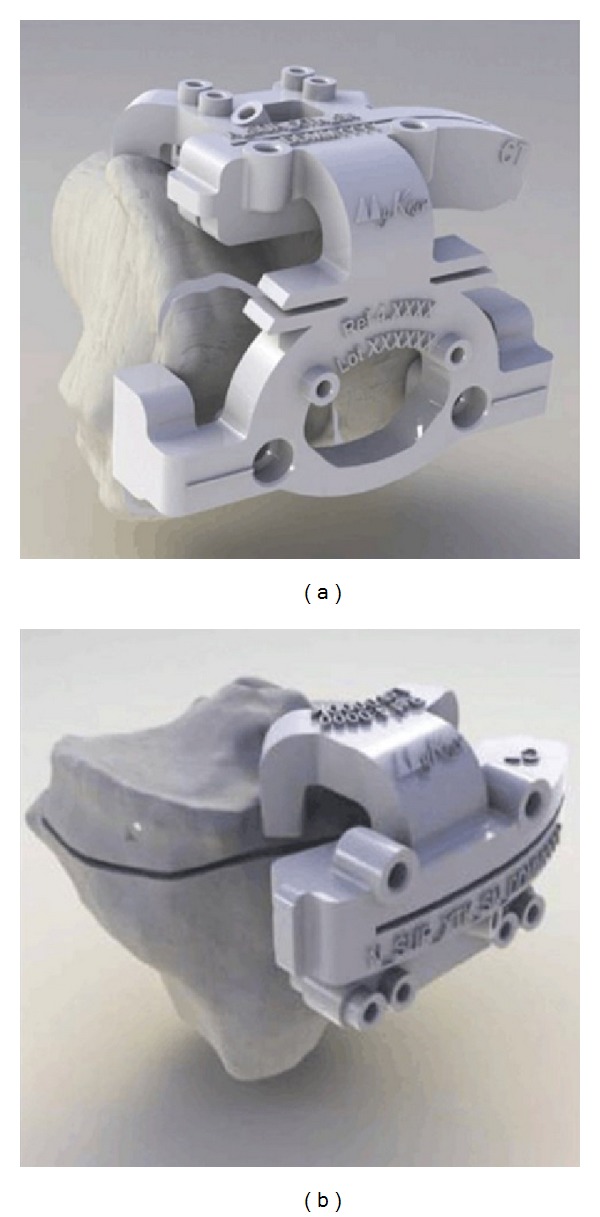
Company manufactured patient specific cutting blocks, mounted on the respective models of the distal femur (a) and proximal tibia (b). Planned cuts are marked on the models.

Models from the proximal tibia and distal femur of each patient are delivered with the PSCBs. They show on which bony landmarks/osteophytes the PSCB needs to be seated (Figure 4). Any cartilage needs to be removed without damaging the osteophytes because they serve as landmarks for the correct seating of the PSCBs.

**Figure 5:**
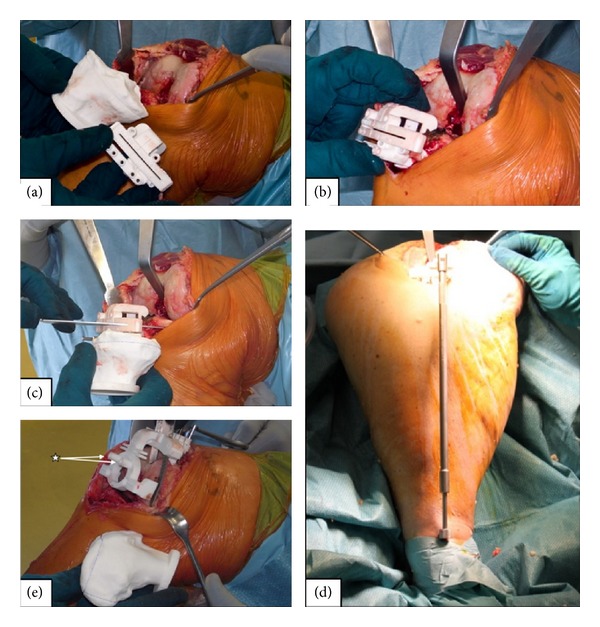
Key steps in PSCB technique: the tibial contact points are identified with the help of the cutting block in conjunction with the model (a). The tibial cutting block is adapted to the contact points on the patient's tibia (b). If optimal fitting is achieved, the resection plane through the cutting block is compared with the one drawn at the tibia model (c). Rotational alignment is controlled with the extramedullary telescopic rod (d). When correct cutting block position is achieved, it is secured to the proximal tibia and the tibial cut is performed. For the femoral cut, the distal femur is exposed and soft tissue/cartilage covering the landmarks is removed. The PSCB is positioned on the patient's femur and fixed before the distal femoral cut is performed (e). Femoral rotation is determined with drilling of two anterior referencing pin holes through the cutting block (asterisk) before performing the femoral cut.

In our practice, we start with the tibial cut, but this is the surgeon's choice. The tibial and femoral cutting blocks are placed on the osteophyte contact points and the resection planes can be compared to the tibial and femoral models (Figures 4 and 5). An extramedullary telescopic rod is used to control the correct tibial alignment in situ. Corrections in tibial height and femoral resection and rotation can be done at any point of the surgery. Femoral and tibial finishing is done as described in the surgical technique for the standard primary GMK TKA. Implantation of the final components depends on whether cemented or noncemented implants are used and is similar to standard technique.

## 3. Results

### 3.1. Coronal Alignment

Preoperatively, only 20.4% of patients had a mechanical axis within 3° of the normal value. A maximum varus angulation of 22.5° and a maximum valgus angulation of 19.7° were noted. Hereby, the larger deformities seemed to occur on the tibial side (Table 1).

Table 2 shows the mean differences in the alignment parameters before and after surgery.

Concerning the HKA as parameter for good mechanical axis alignment, the proportion of patients with a deviation of ≤3° of normal was 81.4% postoperatively and for DFA and PTA 94.7% and 92.0%, respectively. If the margin of error is relaxed to a maximum deviation of 4° from normal, the proportion of patients within this limit is 92.9% for HKA and 99.1% for DFA and 98.2% for PTA (Tables 3, 4, and 5).

In our dataset, the success rate for coronal alignment is higher for the single component position than for the overall mechanical leg axis represented by HKA. When considering all coronal alignment parameters, we found 79.6% of patients within the 3° limit. The parameter in which most of the outliers exceeded the 3° limit was the HKA with optimal values in the other two coronal parameters for the same patient. Only two patients showed a suboptimal alignment of the femoral component and two additional patients a combination of varus femoral position and varus mechanical axis.

**Figure 6:**
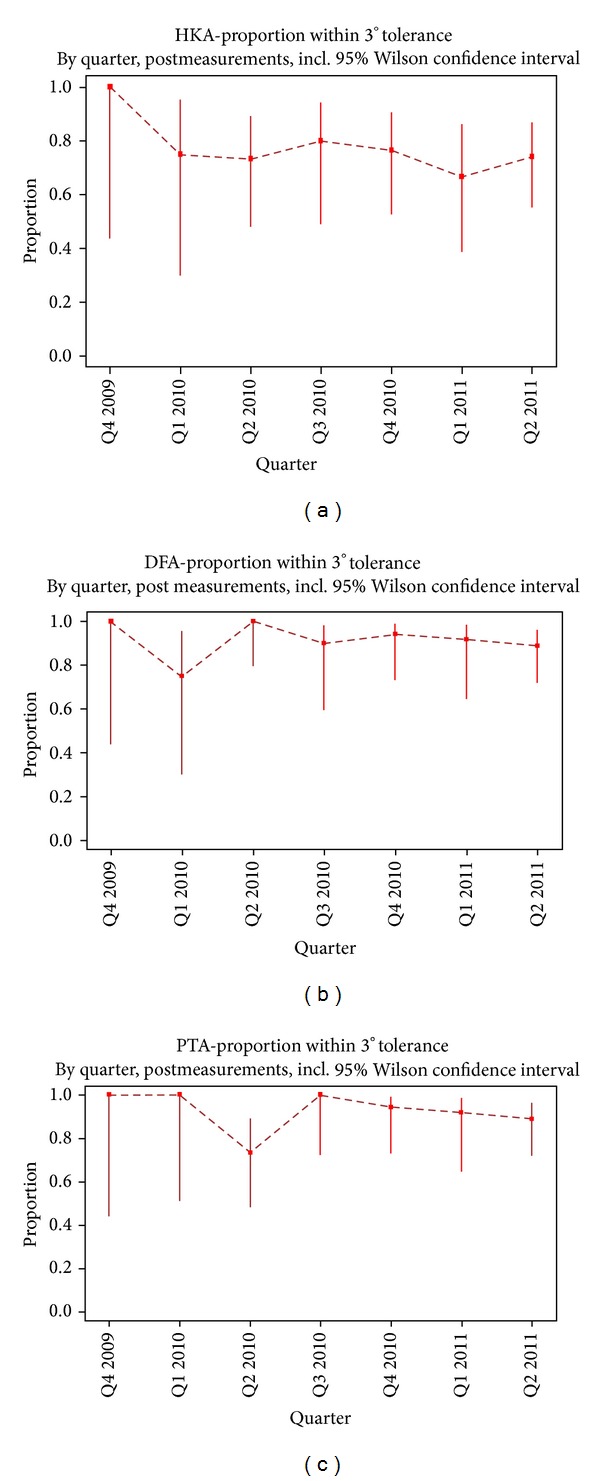
Proportion of patients within the 3° deviation for all alignment parameters over time. No significant changes could be observed.

There was no significant change in the accuracy of the postoperative mechanical leg alignment over time (Figure 6), which suggests that this technique has a steep learning curve.

### 3.2. Sagittal Alignment

Concerning the sagittal alignment represented by the tibial slope, we observed preoperatively a mean tibial slope of 4.62° ± 2.76° with a range of −9° to 10.5°. Negative prefix indicates a tibial plateau that ascends in the posterior direction. Postoperatively, the mean posterior tibial slope was 2.86° ± 2.02° with a range of −4.3° to 9°.

Between the pre- and the postoperative values for TS, we found a mean difference of −1.76° ± 2.85°.

Hypothesizing that the goal of surgery is to maintain the individual posterior tibial slope and to change it no more than 3° in each direction, this goal was achieved in 78.8% of our patients.

Out of the 24 outliers in the TS measurement, ten patients showed a relatively high preoperative posterior slope of more than 7°, what was corrected to values between 1.5° and 7°.

### 3.3. Operating Time

A mean operating time of 95 minutes (range 49–140 minutes, SD 19) was found. When dividing into subgroups according to the experience level (very experienced (*n* = 70), lesser experienced (*n* = 22), and teaching operations (*n* = 17)), mean operating times of 88 minutes (95% CI 84.6–95.1), 106 minutes (95% CI 99.3–113), and 95 minutes (95% CI 81.5–108.2) have been recorded.

**Figure 7:**
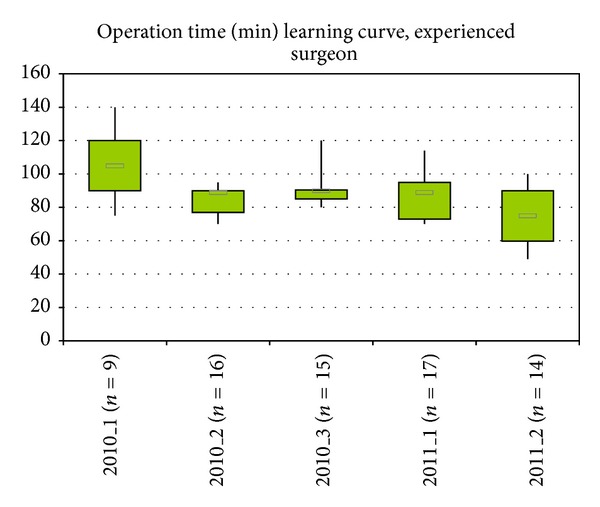
Development of operation time of the experienced surgeon (NH) over time.

In means of a learning curve, there was tendency to shorter operating times on later performed surgeries within the cases of the experienced surgeon (Figure 7). In the other two subgroups, there was no tendency calculated due to the low number of observations.

## 4. Discussion

The presented study suggests that component orientation in total knee arthroplasty can be improved using patient specific cutting blocks.

Superior long-term results, better mechanical wear patterns, and lower failure rates can be obtained by the restoration of a neutral mechanical axis in TKA [2–8].

These results can be achieved by CAS [19]. Increased OR time, the need for additional technical equipment or the learning curve are potential downsides of this technique [20–23]. With this in mind, PSCBs were developed to take full advantage of the accuracy of the computer navigation while suppressing its flaws.

However, in conventional series, the proportion of outliers from ideal alignment (±3°) lies between 2.0% and 72.0%. Comparable CAS-series show a percentage of outliers between 0% and 28,8% [10–12, 24, 25].

In our series, we achieved a coronal mechanical alignment within the deviation of 3° concerning HKA, DFA, and PTA in 81.4%, 94.7%, and 98.2% of cases, respectively. Compared with the current literature, these results are not superior nor inferior to the CAS results but superior to conventional techniques. When looking at the results more in detail, it is obvious that the coronal single component alignment showed very good results, whereas the HKA showed minor results to comparable studies concerning success rates in CAS-series. A literature review of 29 quasirandomized/randomized controlled trials and 11 prospective comparative studies (CAS versus conventional) revealed the proportion of HKA with a margin of error of 3° of 88.9% in the CAS-series as well as proportions for DFA and PTA of 94.1% and 95.5%, respectively [26].

The mismatch between the inferior values for mechanical leg alignment (HKA) and the ideal values for single component alignment is more likely caused by an incorrect indication/decision intraoperatively than caused by the surgical technique. As this deviation in HKA is only seen in long-leg standing (weight-bearing) radiographs, it is more likely caused by a ligament insufficiency that causes dynamic instability. A constrained or hinged type of implant would have been the better choice in these cases.

Concerning the relatively new patient specific instrumentation, only one retrospective review with a larger number of patients was published by Ng et al. [27]. Other than in our study, the surgeons worked with MRI-based patient specific positioning guides (PSPG) that require additional surgical steps and without the use of tibial/femoral models to control the cuts. Ng reviewed 160 patients (105 PSPG-technique and 55 conventional). The postoperative mean HKA was shown to be 0.6° in the PSPG group, compared to our mean of 0°. Concerning the proportion of patients within the margin of error of 3°, they reported superior results with 91.0% compared to 81.4% in our group. For component alignment, they set the margin of error on ±2° and achieved the same results for DFA with 78% and superior results for the PTA with 90% compared to 83.0% in our group (Table 3).

There was no information regarding sagittal alignment or surgical time in this study.

Other investigators of this technique showed good coronal alignment in plastic and cadaveric knees [28, 29]. Concerning the clinical application of the method, only two other studies are published. Klatt et al. evaluated in a case series of four patients the PSCB-recommended cuts with a navigation system and showed big deviations in coronal and sagittal planes from ideal values [30]. Spencer et al. implanted custom-fit knee prosthesis in 21 patients and evaluated the postoperative mechanical alignment with scanograms. 9.5% showed a deviation of more than 3° of ideal alignment concerning HKA, mean alignment was 1.2° ± 2.4 varus, mean deviation of neutral femoral component alignment was 1.6° ± 1.8° valgus, and mean deviation of neutral tibial component position was a varus position of 2.9° ± 2.1°. Sagittal alignment has not been evaluated [31].

Concerning the posterior tibial slope (TS), we noted a slight decrease in mean values from 4.62° to 2.86°. Goal of surgery concerning the TS is much less defined in current literature than the coronal alignment and different options to set the goal of surgery exist.

Choosing the “appropriate slope” is one option whereas the appropriate slope is not well known. Maintaining the patients slope is another option [32], but the slope has a very high interindividual variability and differs between different subsets of populations [33, 34]. The third option is to aim the slope to the value that is proposed by the implant-manufacturing company. There is no consensus concerning the optimal slope and it is known that mechanical effects of different TS depend on the prosthesis design and type (CR versus PS) as well as the choice of the inlay (flat, curved, and posterior lipped). Depending on these factors, the amount of femoral rollback and anteroposterior femorotibial translation differs [35]. An increasing anteroposterior translation which occurs with CR-designs and decreased tibial slope can result in accelerated polyethylene wear [36, 37].

Kinematic in vitro and in vivo studies showed that increasing the TS leads to more laxity not only in the anteroposterior direction but also in rotational and mediolateral directions [38, 39]. Thus, it is beneficial if the knee is tight in flexion intraoperatively. Increased slope produces a more posterior located femorotibial contact area [40].

A higher range of motion with more TS was shown in biomechanical and clinical studies; others showed no influence. Long-term results on different prosthetic designs and failure rates due to loosening and wear depending on the slopes are absent in current literature [35, 41–43].

Studies supporting the option of maintaining the preoperative existing TS rely mainly on the observation that a cut parallel to the tibial plateau and perpendicular to the trabeculae may result in better bone resistance, leading to a smaller risk of tibial component subsidence [32, 40, 44]. This seems to be reasonable, but biomechanical implications of TS on prosthetic wear and ROM as described above should be taken into consideration too.

Therefore, there is no evidence or target value for TS in TKA. Most prosthetic manufacturers suggest values between 0 and 3°; many authors suggest values of 0–7°. Literature only agrees to the fact that a reverse TS should be avoided.

Our results for TS are comparable with the ones from CAS-studies but the clinical value of this remains unclear. In comparative studies between CAS and conventional TKA, CAS seems to be superior to conventional technique concerning TS. Jenny and Boeri compared both techniques and showed that 85% of the CAS patients versus 70% of the conventional patients reached the goal of surgery concerning TS [24]. Significant improvement of postoperative sagittal tibial alignment could be shown in other CAS-studies [45]. Our results are slightly inferior to the CAS results but clearly superior to conventional results achieving optimal alignment in 78.8% and 85.0% when predicting a margin of error of 3° or 4°, respectively.

Concerning patient specific instrumentation only, the most recent study evaluated the sagittal tibial alignment too [46]. Mean deviation was 1.16° ± 4.29° (range of −4.5° to 9.0°). Proportion of patients within a maximal deviation of 2° was 40.0%, compared with 65.5% in our study.

In means of alignment, there was no learning curve. Accuracy was unchanged over the whole period for all groups of surgeons. Interestingly, there has been a difference between the very experienced and the lesser experienced surgeon which leads to the hypothesis that accuracy depends less on the technique itself than on the overall surgical experience. This would mean that in the hands of an experienced surgeon PSCB is a well-functioning instrument right from the beginning but, at the same time, that experience is an important factor even with PSCB and that PSCB cannot replace experience and understanding of total knee arthroplasty.

Contradictory to accuracy, a high variability (range from 49–140 minutes) and a learning curve have been observed concerning the operating time. A decrease from an average of 105 to 77 minutes was detected for the experienced surgeon within 14 months. The biggest decrease in the average time happened between the first and the second 4-months-period suggesting a steep learning curve.

Compared with operating times and learning curves published in current literature, it could be shown that, in CAS groups, the mean surgical time was not significantly decreasing over time. Even when the surgeon gets familiar with the system, the operating time is not decreasing. This is due to the fact that in CAS several operating steps need to be done, whereas the PSCB-technique decreases the number of operating steps to a minimum. On the other hand, studies showed that surgeons not familiar with computer-assisted surgery/navigation achieved a significantly lower accuracy [11, 47, 48].

## 5. Conclusion

We presented the radiological results of our first 113 TKA-patients, operated with patient specific cutting blocks. This technique seems to achieve a good accuracy, comparable with CAS techniques, and clearly superior to conventional techniques. The learning curve is steep and even residents are able to achieve very good results in terms of mechanical alignment. Advantages compared to CAS include no implantation of additional pins for trackers and thus less pin-associated complications. Additionally, less surgical steps are needed as well as less instrumentation because implant sizes are calculated preoperatively. This has the potential to reduce not only OR time but also sterilization costs.

## Figures and Tables

**Table 1:** Summary of the minimum, maximum, mean, and standard deviation of the tested variables (note that negative prefix means a varus angulation and positive prefix a valgus angulation).

Variable	Time point	Mean	Min	Max	STD
HKA	Before	−2.8	−22.5	19.7	9.7
	After	−0.2	−9.6	5.8	2.6

TS	Before	4.6	−9.0	10.5	2.8
	After	2.9	−4.3	9.0	2.0

DFA	Before	88.6	80.0	97.3	3.6
	After	89.8	86.4	96.0	1.7

PTA	Before	87.8	76.7	101.0	4.8
	After	89.9	84.3	93.8	1.7

**Table 2:** Differences between pre- and postoperative values (note that negative prefix means a varus angulation and positive prefix a valgus angulation).

Variable	Mean	Min	Max	STD
HKA	2.61	−20.1	19.2	8.95
TS	−1.76	−8.2	9.0	2.85
DFA	1.21	−9.2	9.1	3.73
PTA	2.07	−13.0	15.2	4.97

**Table 3:** Proportion of patients within the different margins of error for the coronal alignment parameter HKA.

Variable	Time point	Deviation	Proportion of patients in norm	95% Wilcoxon CI
HKA	Before	1	8.8%	[0.05, 0.16]
	After	1	35.4%	[0.27, 0.45]
	Before	2	15.0%	[0.10, 0.23]
	After	2	61.9%	[0.53, 0.70]
	Before	3	20.4%	[0.14, 0.29]
	After	3	81.4%	[0.73, 0.88]
	Before	4	26.5%	[0.19, 0.35]
	After	4	92.9%	[0.87, 0.96]

**Table 4:** Proportion of patients within the different margins of error for the coronal alignment parameter DFA.

Variable	Time point	Deviation	Proportion of patients in norm	95% Wilcoxon CI
DFA	Before	1	18.6%	[0.12, 0.27]
	After	1	48.7%	[0.40, 0.58]
	Before	2	42.5%	[0.34, 0.52]
	After	2	78.8%	[0.70, 0.85]
	Before	3	54.9%	[0.46, 0.64]
	After	3	94.7%	[0.89, 0.98]
	Before	4	65.5%	[0.56, 0.74]
	After	4	99.1%	[0.95, 1.00]

**Table 5:** Proportion of patients within the different margins of error for the coronal alignment parameter PTA.

Variable	Time point	Deviation	Proportion of patients in norm	95% Wilcoxon CI
PTA	Before	1	17.7%	[0.12, 0.26]
	After	1	56.6%	[0.47, 0.65]
	Before	2	31.9%	[0.24, 0.41]
	After	2	82.3%	[0.74, 0.88]
	Before	3	44.2%	[0.35, 0.53]
	After	3	92.0%	[0.86, 0.96]
	Before	4	57.5%	[0.48, 0.66]
	After	4	98.2%	[0.94, 1.00]
